# Cough Is Dangerous: Neural Correlates of Implicit Body Symptoms Associations

**DOI:** 10.3389/fpsyg.2016.00247

**Published:** 2016-03-01

**Authors:** Daniela Mier, Michael Witthöft, Josef Bailer, Julia Ofer, Tobias Kerstner, Fred Rist, Carsten Diener

**Affiliations:** ^1^Department of Clinical Psychology, Central Institute of Mental Health, Medical Faculty Mannheim, University of HeidelbergGermany; ^2^Department of Clinical Psychology, Psychotherapy and Experimental Psychopathology, Johannes Gutenberg UniversityMainz, Germany; ^3^Department of Clinical Psychology, University of MünsterGermany; ^4^Department of Cognitive and Clinical Neuroscience, Central Institute of Mental Health, Medical Faculty Mannheim, University of HeidelbergGermany; ^5^School of Applied Psychology, SRH University of Applied SciencesHeidelberg, Germany

**Keywords:** health anxiety, implicit association test, functional magnetic resonance imaging, prefrontal cortex, executive functions

## Abstract

The negative interpretation of body sensations (e.g., as sign of a severe illness) is a crucial cognitive process in pathological health anxiety (HA). However, little is known about the nature and the degree of automaticity of this interpretation bias. We applied an implicit association test (IAT) in 20 subjects during functional magnetic resonance imaging (fMRI) to investigate behavioral and neural correlates of implicit attitudes toward symptom words. On the behavioral level, body symptom words elicited strong negative implicit association effects, as indexed by slowed reaction times, when symptom words were paired with the attribute “harmless” (incongruent condition). fMRI revealed increased activation in the dorsolateral prefrontal cortex (DLPFC) and posterior parietal cortex for the comparison of incongruent words with control words, as well as with a lower significance threshold also in comparison to congruent words. Moreover, activation in the DLPFC, posterior parietal cortex, nucleus accumbens, and cerebellum varied with individual levels of HA (again, in comparison to control words, as well as with a lower significance threshold also in comparison to congruent words). Slowed reaction times as well as increased activation in dorsolateral prefrontal and posterior parietal cortex point to increased inhibitory demands during the incongruent IAT condition. The positive association between HA severity and neural activity in nucleus accumbens, dorsolateral prefrontal, and posterior parietal cortex suggests that HA is characterized by both intensified negative implicit attitudes and hampered cognitive control mechanisms when confronted with body symptoms.

## Introduction

Automatic biases in information processing seem characteristic for individuals suffering from mental disorders (American Psychiatric Association, 2000, 2013). Disorder-related external or internal stimuli appear to be processed with high priority on an automatic ‘route’ (e.g., Barrett et al., 2004; Strack and Deutsch, 2004), hardly controllable by the patients. In pathological health anxiety (HA), for instance, normally negligible body sensations (e.g., a momentary tremor in a muscle) can trigger catastrophic worries about suffering from a severe illness (e.g., amyotrophic lateral sclerosis; Weck et al., 2011).

To objectively assess the strength of such automatic associations in memory, the implicit association test (IAT; Greenwald et al., 1998) has been successfully applied in several mental disorders. IAT findings in anxiety disorders (Ellwart et al., 2006; Westberg et al., 2007; Teachman et al., 2008) including post-traumatic stress disorder (Engelhard et al., 2007), addiction (Wiers et al., 2002), somatoform disorders (Buhlmann et al., 2009; Weck et al., 2011; Riebel et al., 2013), and also borderline personality disorder (Rüsch et al., 2007) consistently emphasize the significance of disorder-related implicit associations which seem closely tied to negative affect as well as dysfunctional cognitive and behavioral control (e.g., avoidance or defensive behavior and/or impulsive acts).

However, studies investigating the neural basis of altered implicit associations in clinical samples using the IAT are missing. With regard to the neural correlates of IAT performance, neuroimaging findings have been obtained for the original procedure and adapted versions mainly in social prejudice research (for a review, see Beer et al., 2008). In the original IAT, two categories of target words (e.g., flowers and insects) and two categories of attributes (e.g., pleasant and unpleasant) have to be classified simultaneously using two response buttons. By altering the combination of targets and attributes to the two response buttons, a congruent (e.g., flowers/pleasant; insects/unpleasant) and an incongruent condition (e.g., flowers/unpleasant; insects/pleasant) is constructed. Significantly slower response times in the incongruent condition as compared to the congruent condition are regarded as evidence for an implicit bias toward congruent associations between targets and attributes. However, this behavioral outcome may inherently represent the joint effect of automatic (implicit) and controlled processes (Beer et al., 2008): Strong biases can result from strong implicit evaluative associations and/or difficulties to inhibit these as required during the incongruent condition. Hence, many different theoretical accounts have been proposed to explain IAT effects in terms of salience asymmetries between concepts (Rothermund and Wentura, 2004) or differences in executive functions such as task switching (Mierke and Klauer, 2001). Consequently, IAT scores should best be interpreted as an amalgam of different processes associated with concept activation and attempts to overcome incongruence (Sherman et al., 2008). Chee et al. (2000) found for the original ‘insect–flower’ IAT increased activity in the dorsolateral prefrontal cortex (DLPFC) and the anterior cingulate cortex (ACC) in the incongruent condition, suggesting that these prefrontal regions were involved in the detection and resolution of cognitive conflict (Etkin et al., 2006) and inhibition of pre-potent responses (Kadota et al., 2010). Increased activity in the DLPFC was also found when healthy subjects were required to inhibit responses coupled with implicit beliefs about gender and race (Knutson et al., 2007). Likewise, Beer et al. (2008) found increased activity in the DLPFC and ACC during conflicting response tendencies linked to social attitudes. In contrast to converging results for especially the DLPFC (and ACC) to be involved in controlled IAT demands (incongruent condition), several approaches to isolate brain regions linked to implicit, automatic mechanisms (congruent condition) found a variety of areas which seem to mediate in particular the processing of the emotional significance of stimulus materials. These areas include (orbitofrontal and medial) prefrontal regions, the insula, and limbic (e.g., amygdala) including striatal (e.g., caudate) structures (Luo et al., 2006; Knutson et al., 2007; Beer et al., 2008). Thus, it seems reasonable to assume that IAT effects as found on the behavioral level mainly represent the joint contribution of a bottom-up network consisting of orbitofrontal/medial prefrontal, insular, and limbic-striatal regions, which seem predominately linked to implicit mechanisms of initial stimulus evaluation, and the DLPFC (and ACC) being involved in top-down control of response conflicts when stimulus response contingencies mismatch the affective evaluation [for a detailed sub-process (Quad) model of automatic and controlled processes, see Beer et al., 2008].

However, most of the previous studies aiming to disclose the neural correlates of automatic mechanisms involved in IAT performance used an indirect approach (Cunningham et al., 2004; Phan et al., 2004). These studies used correlational analyses between IAT scores measured before scanning and neural activation during the confrontation with the stimuli of interest (e.g., black and white faces). This approach assumes inter-individual differences in IAT scores to reasonably covary with inter-individual differences in neural activation in a different but related task, which has been shown to be effective in activating brain regions generally assumed to be involved in implicit appraisal mechanisms of affective stimuli. Although methodologically sound, this approach affords no information about the factual neural activity during IAT performance. Thus, we propose to apply the IAT directly during functional neuroimaging in subjects which differ in the extent of the attitude of interest.

To this aim, HA may represent an excellent psychological condition for the study of the neural correlates of the IAT in the context of mental disorders. Excessive anxiety and constantly worrying about one’s health is characteristic of patients with the diagnosis of hypochondriasis (American Psychiatric Association, 2000). In DSM-5, hypochondriasis was split into two novel diagnoses: individuals with high HA now receive the diagnosis somatic symptom disorder if they suffer from an additional somatic symptom, or the diagnosis of illness anxiety disorder, if they do not experience distressing somatic symptoms (Bailer et al., 2015).

Moving beyond a clinical dichotomous perspective, HA is viewed as a dimensional construct ranging from absent health concerns to pathological HA (Ferguson, 2009). Cognitive-behavioral (Warwick and Salkovskis, 1990) and cognitive-developmental (Williams, 2004) as well as interpersonal models (Noyes et al., 2003) of HA stress the importance of illness-related schemata in memory that developed in childhood and adolescence and may become triggered by current instances in later life and guide information processing in dysfunctional ways (Marcus et al., 2007). Probably the most important of these cognitive processes represent the catastrophic (mis)interpretation of body sensations/symptoms as a sign of a severe illness. However, experimental research on this ’interpretation bias’ is relatively sparse since most research solely focused on self-report data (Marcus et al., 2007). Moreover, it is an open question which brain regions are dominantly involved in biased processing of body symptoms in HA.

Against this background, the current study pursued two goals: (1) to validate a HA variant of the IAT measuring implicit attitudes (“dangerous” vs. “harmless”) to body symptoms on the behavioral level and (2) to disclose the neural correlates of the HA IAT in healthy subjects differing in the extent of HA. When focusing on the incongruent condition, in line with prior neuroimaging IAT findings, we mainly expected increased activity in the DLPFC representing enhanced effortful mechanisms to inhibit implicitly biased response tendencies. In contrast to this main effect expected to be present across differences in the individual extent of HA, we hypothesized that activity in areas proposed to be linked to implicit mechanisms of stimulus evaluation (orbitofrontal/medial prefrontal, insular, limbic-striatal regions) is related to the extent of HA. However, based on the assumption that these regions interact with the DLPFC during the incongruent condition, we expected the DLPFC to additionally show incremental activity linked to the extent of HA.

## Materials and Methods

In the following, we report all data exclusions, all manipulations of the data, and all measures used in the study.

### Participants

Twenty healthy individuals (11 females) were included in the study (*M* age ± *SD*: 22.8 ± 2.22 years; range: 20–27 years). Four more subjects participated, but had to be excluded due to excessive head movement (translation > 2 mm), and four participants due to technical problems (responses were not recorded in two subjects and data quality was low in the other two subjects; i.e., no signal variation in the visual cortex, as well as in the motor cortex in the symptom words condition, even at a low threshold of *p* < 0.5). A minimum of 20 participants was assumed as sufficient to allow correlation analyses. All participants were right-handed and had normal or corrected-to-normal vision. Before participating in the study subjects gave their written informed consent. The study was approved by the Medical Ethics Committee of the Medical Faculty Mannheim, University of Heidelberg, Germany and conducted in concordance with the declaration of Helsinki. All participants completed the Whitley Index (WI; German version: Hiller and Rief, 2004) as a dimensional measure of HA (*M* ±*SD*: 2.8 ± 2.4; range: 0–7). None of the participants reached the clinically relevant cut-score of WI ≥ 8 that is used in clinical studies to identify people with full-blown hypochondriasis (Hiller et al., 2002).

### Procedure

For the sake of completeness, it should be mentioned that additionally to the procedures reported in the following, participants also completed the somatic symptom index PHQ-15, as well as the PHQ-9 of the Patient Health Questionnaire (Spitzer et al., 1999), and performed an emotional stroop task (Witthöft et al., 2013).

#### Self-Report Measures

The Whitley-Index (WI) was used to assess the extent of HA. The WI is a widely applied 14-item instrument with a dichotomous response format for the dimensional assessment of hypochondriacal concerns (sample item: Do you worry a lot about your health?). The reliability, validity, and specificity of the WI for the assessment of HA have been demonstrated (Hiller and Rief, 2004). Cronbach’s α coefficient in the study sample was 0.73.

#### Implicit Association Task (IAT)

The symptom-word IAT used in this study is based on a previous task version that was developed and piloted in a study on cognitive mechanisms of HA among college students (Schmidt et al., 2013). In the current symptom IAT, 10 body symptom words referring to common bodily complaints or sensations (e.g., dizziness, headache, nausea) considered as potential triggers of illness concerns in patients with hypochondriasis (Barsky et al., 1993) and 10 neutral words (e.g., toaster, bowl, plate) serve as target stimuli. The 10 target words per category were already used in a study on attentional bias in HA [Witthoft et al., 2008; in the original IAT-task version (Schmidt et al., 2013) only five target word stimuli in each category have been used]. The concepts “harmless” and “dangerous” served as attribute dimension (**Table 1**), and the words “symptom” and “household” as category labels. Target words were randomly presented in blocks, alternating with control task blocks, which showed rows of lower case and capital letters, as presented by Chee et al. (2000). Subjects had to assign target words to one of four categories, depicted left and right on the upper half of the screen. With these stimuli congruent and incongruent conditions were operationalized. All participants started with the congruent condition, wherein symptoms and adjectives of the category “dangerous” shared the same response button (and household related and “harmless” words, respectively). In the incongruent condition, the positions on the screen (left/right) for the categories “dangerous” and “harmless” were replaced, resulting in a pairing of “harmless” with symptom words and “dangerous” with household words.

**Table 1:** Original and translated symptom and neutral target words used in the implicit association test (IAT).

Original stimuli (in German)		Translated stimuli	
Symptom words	Neutral words	Symptom words	Neutral words
Schwindel	Toaster	Dizziness	Toaster
Übelkeit	Kochlöffel	Nausea	Wooden spoon
Kopfschmerzen	Waschbecken	Headache	Basin
Durchfall	Besteck	Diarrhea	Canteen
Atemnot	Teelöffel	Breathlessness	Tea spoon
Schmerzen	Schüssel	Pain	Bowl
Herzrasen	Handfeger	Tachycardia	Hand brush
Erbrechen	Esslöffel	Sickness	Soup spoon
Bauchschmerzen	Topflappen	Abdominal pain	Oven gloves
Husten	Teller	Cough	Plate

*For the IAT attribute dimensions “harmless” and “dangerous” the following adjectives were used (Harmless: harmless, ordinary, normal, safe, common; Dangerous: dangerous, threatening, risky, catastrophic, fatal).*

In the control condition, rows of lower case or capital letters had to be assigned to the attributes “lower case” or “capital.” To control for possible differences in reversal learning abilities and associated cognitive effort between subjects, the lower case and capital letter categories were also reversed on the screen in the incongruent condition.

Responses (left versus right button press) were given with a Lumitouch optical response device (Photon Control Inc, Burnaby, BC, Canada).

Each experimental block consisted of 16 stimuli, presented at a fixed duration of 1.5 s, followed by an intertrial interval with a fixation cross of a mean duration of 300 ms (i.e., the maximum trial duration and response window comprised 1.8 s). Total duration of each block was 30 s. The experiment started with the congruent conditions (with six word and six letter blocks in alternating order) followed by 12 incongruent conditions with changed classification requirements of words and letters (again six word and six letter blocks in alternating order). A fixation cross was displayed between blocks for 9 s and an instruction for the new block for 1 s. The experiment was implemented by means of Presentation software, version 9.50 (Neurobehavioral Systems, Albany, CA, USA). Stimuli were presented by VisuaStim video goggles (Resonance Technology Inc, Northridge, CA, USA).

#### Image Acquisition

Data acquisition was accomplished via a 1.5 Tesla Siemens Vision whole-body magnetic resonance tomograph (Siemens Medical Systems, Erlangen, Germany). The functional images were acquired with a T2^∗^-weighted gradient echo planar imaging sequence (TR = 3000 ms; TA = 100 ms; TE = 50 ms; flip angle 90°; field of view = 224 mm; 64 × 64 matrix). 29 slices per volume were collected in descending order with a slice-thickness of 5 mm and 1 mm gap (resulting voxel size: 6 mm × 3.4 mm × 3.4 mm). The 189 scans were collected for each session (congruent and incongruent), resulting in a total of 378 scans for the experiment. Prior to the functional imaging a T1-weighted anatomical scan was collected from all subjects (162 slices, 1 mm × 1 mm × 1 mm voxel size).

#### Data Reduction and Statistical Analyzes

fMRI data analysis was done with SPM5 (Wellcome Department of Cognitive Neuroscience, London). Data preprocessing consisted of realignment, spatial normalization (MNI template) and spatially smoothing (8 mm FWHM kernel). A first-level fixed effects analysis was calculated, including both sessions of each person. Regressors for each condition were defined and convolved with a box car function. To minimize the influence of movement related variance, the six movement parameters of the realignment procedure were included as covariates of no interest. Furthermore, to control for possible noise in the data, time series from the white substance and the ventricles were extracted and also included as covariates. In second-level random effects analyses individual contrasts from the first-level were taken together for the group analysis [congruent words > congruent letters; incongruent words > incongruent letters; (incongruent words > incongruent letters) > (congruent words < congruent letters)]. Voxel-wise significance threshold for the analyses was set to *p* < 0.001. In addition, to control for multiple testing, cluster size thresholds were assessed with alphasim (http://afni.nimh.nih.gov/afni/), depending on the smoothness of the data (i.e., the estimated spatial intercorrelation of the data, depending on the noise distribution) for each analysis separately, allowing a significance threshold of *p* < 0.05 for false-positives. In addition, all analyses were conducted on a more lenient significance threshold of *p* < 0.005, *k* = 10 (Lieberman and Cunningham, 2009). Main effects of condition were investigated with *t*-tests. Additional regression analyses were performed to investigate the association between HA (*WI*-score), as well as between the *D*_4_ score and brain activation in the incongruent condition [incongruent words > incongruent letters; (incongruent words > incongruent letters) > (congruent words < congruent letters)].

RT data of the IAT were subjected to a 2 × 2 mixed analysis of variance (ANOVA) with content (words vs. letters) and congruency (congruent vs. incongruent condition) as repeated measurement factors. Significant main and interaction effects were analyzed *post hoc* by means of paired *t*-tests.

As suggested by Greenwald et al. (2003), we additionally computed *D*-scores as a measure of IAT performance to combine response speed and accuracy information. *D*-scores (more precisely *D*_4_ scores) were derived by recoding single RTs of erroneous responses by the individual RT mean plus 600 ms. A RT difference score was computed between the incongruent and the congruent condition and this difference score was divided by the individual overall *SD* of RT data. Please note that for the control task condition (with lower case and capital letters), the *D*-score does not represent a measure of strength of associations but rather an index of switching costs that result from the reversal of the response categories.

We decided to report both, IAT effects based on RT difference scores as well as IAT effects based on the *D*-score for reasons of transparency and because the *D*-score has been subject to theoretical and methodological criticism (e.g., Wentura and Rothermund, 2007). In order to detect possible associations between behavioral IAT effects and the individual level of HA, correlations were computed between the IAT *D*-values and the WI. Behavioral data were analyzed with SPSS Version 13.0 (SPSS Inc., Chicago, IL, USA).

## Results

### Behavioral Data

Participants needed on average 769.62 ms (*SD* = 95.13) in the incongruent word condition, 688.98 ms (*SD* = 87.83) in the congruent word condition, 480.91 ms (*SD* = 58.48) in the incongruent letter condition, and 483.60 ms (*SD* = 65.73) in the congruent letter condition. Corresponding accuracies were 93.1% (*SD* = 3.89) and 91.4% (*SD* = 4.93) in the congruent and incongruent word condition; and 97.4% (*SD* = 2.5) and 85.0% (*SD* = 5.6) for the congruent and incongruent letter condition, respectively.

Results for the mixed ANOVA of the RT data revealed significant main effects for both content [*F*(1,19) = 297.04, *p* < 0.01, $\eta_{p}^{2}$ = 0.94] and congruency [*F*(1,19) = 30.81, *p* < 0.01, $\eta_{p}^{2}$ = 0.62] as well as a significant interaction between content and congruency [*F*(1,19) = 36.78, *p* < 0.01, $\eta_{p}^{2}$ = 0.66]. *Post hoc t*-tests indicated that responses to letters were significantly faster compared to words [*t*(19) = 17.24*, p* < 0.01; *d* = 3.35] and that responses were faster in the congruent compared to the incongruent condition [*t*(19) = 5.55, *p* < 0.01; *d* = 0.56]. *Post hoc* tests within each condition (words and letters) revealed that only in the word condition [*t*(19) = 7.46, *p* < 0.01; *d* = 0.88] but not in the letter control condition [*t*(19) = –0.31, *p* = 0.76; *d* = –0.04] a significant congruency effect was present.

The mean *D*-score was 0.36 (*SD* = 0.23) for the IAT word task and 0.35 (*SD* = 0.26) for the letter control condition. A paired *t*-test of the two *D*-scores showed no significant difference between the two tasks [*t*(19) = 0.20, *p* = 0.84; *d* = 0.06].

There was no significant correlation between the extent of HA (*WI*-score) and the *D*-score (*r* = 0.11, *p* = 0.64). As expected, in the control condition (letters) no significant association to the WI occurred.

### Functional Imaging Data

**FIGURE 1:**
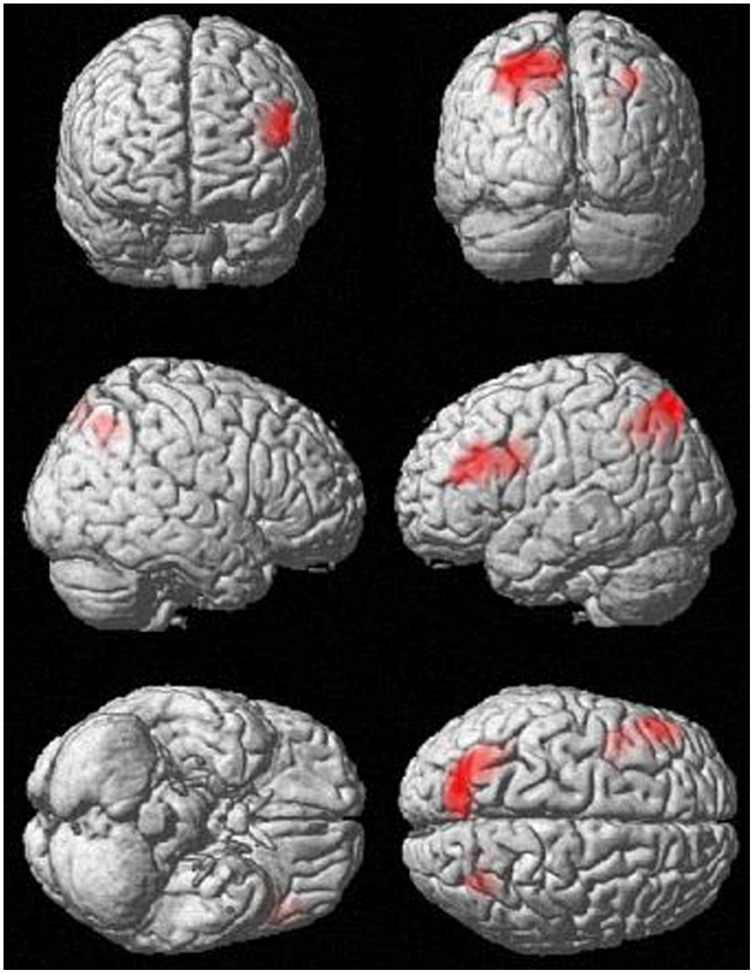
**Activation in the incongruent condition (incongruent words > incongruent letters), *p* < 0.001, *k* > 83**.

Analyses of fMRI data revealed significant results for the incongruent word condition (see **Table 2** and **Figure 1**). During the incongruent word condition as compared to the incongruent letter condition, subjects showed enhanced activation in the left DLPFC and bilaterally in the posterior parietal cortex.

**Table 2:** Activation in the incongruent condition (incongruent words > incongruent letters).

				MNI			
Area	BA	L/R	Cluster	*x*	*y*	*z*	*t*-value
**Main effect**							
Dorsolateral prefrontal cortex	46	L	389	-48	27	27	5.97
Dorsolateral prefrontal cortex	46	L		-45	39	18	5.04
Posterior parietal cortex	7	L	486	-24	-72	54	6.70
Posterior parietal cortex	7	L		-30	-60	45	6.23
Posterior parietal cortex	7	R	103	21	-63	33	5.20
Posterior parietal cortex	7	R		33	-66	48	5.17
**Positive correlation with WI**							
Superior Temporal Gyrus	22	R	301	66	-39	12	5.45
Supramarginal gyrus	40	R		66	-48	27	4.77
Posterior parietal cortex	7	R		36	-57	60	4.63
Dorsolateral prefrontal cortex	9	R	112	39	18	27	5.42
Dorsolateral prefrontal cortex	46	R		51	30	24	4.65
Caudate Head		L	212	-12	21	-9	4.95
Anterior cingulate cortex	25			0	0	-6	4.31
Caudate head		R		9	15	-6	4.27
Cerebellum		L	87	-30	-33	-33	4.90
Cerebellum		L		-15	-27	-45	4.08
Cerebellum		L		-36	-48	-33	3.78

*Results are reported at a p threshold of p < 0.001 and a corrected cluster size threshold of p < 0.05 (Main effect: Cluster size threshold in voxels: 83; Correlation: Cluster size threshold in voxels 78). WI, Whitley Index.*

**FIGURE 2:**
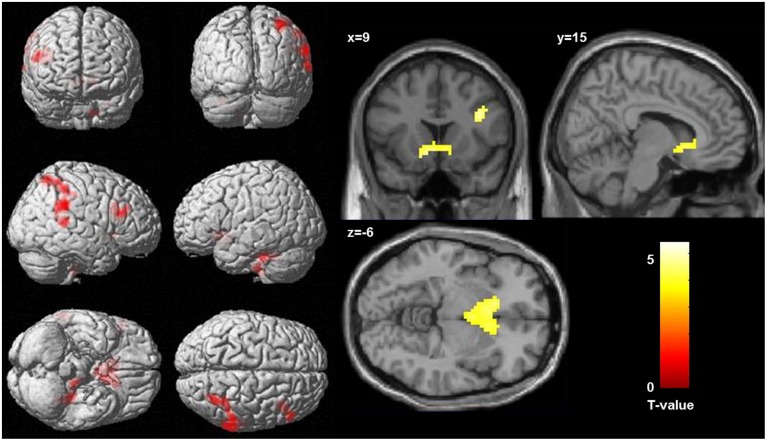
**Correlation between activation in the incongruent condition (incongruent words > incongruent letters) and health anxiety (as measures by the Whitley index), significance threshold is *p* < 0.001, and *k* > 78. (Left)** Display of cortical activation. **(Right)** Display of activation in the nucleus accumbens.

Regression analysis revealed a significant positive correlation between activation in the incongruent condition (incongruent words > incongruent letters) and the *WI*-score (see **Table 2** and **Figure 2**). Higher HA was correlated with bilaterally enhanced activation in the nucleus accumbens reaching into the subgenual ACC, in the right DLPFC, in superior temporal sulcus, reaching into the posterior parietal cortex, and in the left cerebellum.

**FIGURE 3:**
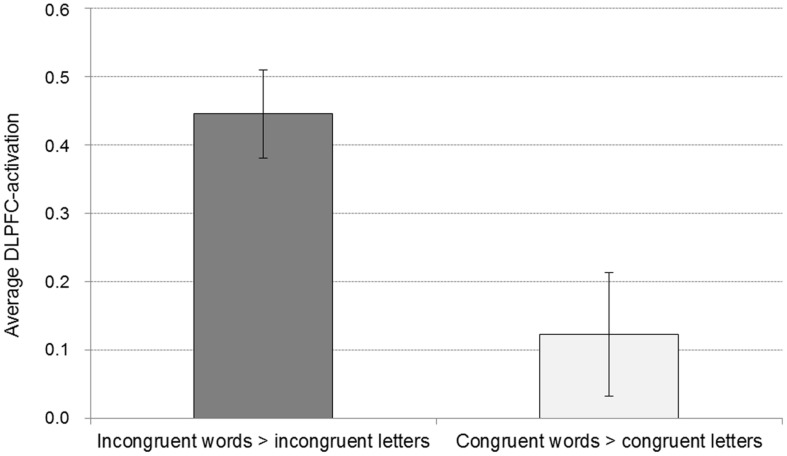
**Average activation for the word conditions in comparison to the respective control conditions of the DLPFC peak voxel of the contrast incongruent words > incongruent letters (with a sphere of 8 mm).** Displayed are means and standard errors.

**FIGURE 4:**
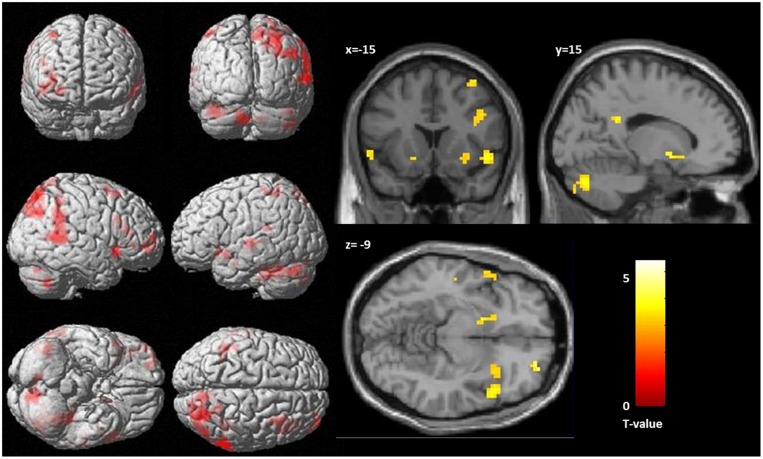
**Correlation between activation, as revealed by the interaction contrast [(incongruent words > incongruent letters) > (congruent words < congruent letters)] and health anxiety (as measures by the Whitley index), significance threshold is *p* < 0.005, *k* = 10. (Left)** Display of cortical activation. **(Right)** Display of activation in the nucleus accumbens.

At the given significance level, results from the analyses of the congruent condition (congruent words > congruent letters) were not significant. The same was true for the comparison of the incongruent and congruent condition with an interaction contrast [(incongruent words > incongruent letters) > (congruent words < congruent letters)]. Also, there was no significant association between the *D*-IAT score and brain activation, in either of the conditions. However, when lowering the significance thresholds to *p* < 0.005, *k* = 10 (Lieberman and Cunningham, 2009), enhanced activation in parietal cortex was found for the interaction contrast for the incongruency condition [(incongruent words > incongruent letters) > (congruent words < congruent letters); **Table 3**], too. Left DLPFC activation for the word conditions in comparison to the letter conditions is displayed in **Figure 3**. However, only 7 voxels were revealed for the left DLPFC, not surpassing the cluster size threshold. Moreover, an association was found between WI and brain activation in the interaction contrast [(incongruent words > incongruent letters) > (congruent words < congruent letters)] in parietal cortex bilaterally, and also in left DLPFC, cerebellum and striatum, as well as additional brain regions that were not found with the more conservative significance threshold for the correlation of incongruent words > incongruent letters and the WI (**Table 3**, **Figure 4**). Furthermore, a significant association was found between the *D*_4_ score and activation in the incongruency condition [(incongruent words > incongruent letters) > (congruent words < congruent letters)] in DLPFC and parietal cortex (**Table 3**). In addition, we found an involvement of parietal and prefrontal cortex for the congruent condition (congruent words > congruent letters), too. These results, however, were all revealed with the more lenient significance threshold (**Table 4**).

**Table 3:** Results from the valence (negative versus neutral) by type (congruent versus incongruent) interaction [contrast: (incongruent words > incongruent letters) > (congruent words < congruent letters)].

				MNI			
Area	BA	L/R	Cluster	*x*	*y*	*z*	*t*-value
**Incongruency effect**							
Posterior parietal cortex	7	L	159	-15	-75	54	3.94
Posterior parietal cortex	7	L		-33	-60	42	3.42
Posterior parietal cortex	7	L		-24	-63	45	3.23
Cerebellum		R	19	36	-72	-54	3.73
Posterior parietal cortex	7	R	15	30	-63	45	3.28
**Positive correlation with WI**							
Posterior Parietal Cortex	7	R	298	30	-72	57	5.11
Angular Gyrus	39	R		51	-72	36	4.27
Posterior Parietal Cortex	7	R		21	-54	75	4.11
Supramarginal Gyrus	40	R	236	66	-48	33	4.90
Superior Temporal Gyrus	22	R		66	-48	24	4.31
Middle Temporal Gyrus	21	R		66	-54	6	4.30
Middle Frontal Gyrus	10	R	21	27	57	-6	4.58
Cerebellum		L	130	-9	-75	-36	4.18
Cerebellum		R		12	-69	-39	3.57
Cerebellum		R		3	-78	-36	2.98
Middle Frontal Gyrus	10	R	21	36	60	6	4.09
Cerebellum		L	256	-33	-51	-33	4.07
Cerebellum		L		-36	-39	-33	3.95
Cerebellum		L		-39	-72	-30	3.82
Cerebellum		R	44	51	-60	-39	4.04
Cerebellum		R		42	-63	-45	3.74
Supramarginal Gyrus	40	L	33	-48	-48	60	3.82
Postcentral Gyrus	2	L		-48	-33	63	3.51
Postcentral Gyrus	5	L		-39	-51	66	3.39
Globus Pallidus		L	14	-12	3	-6	3.68
Putamen		L		-15	15	-9	3.12
Inferior Frontal Gyrus	47	R	43	48	18	-9	3.65
Cerebellum		R	17	45	-72	-30	3.60
Dorsolateral prefrontal cortex	46	R	76	39	33	15	3.57
Dorsolateral prefrontal cortex	9	R		45	15	30	3.25
Inferior Frontal Gyrus	45	R		48	24	27	2.98
Posterior Cingulate	23	L	12	-12	-42	24	3.37
Middle Frontal Gyrus	10	R	28	45	51	-3	3.33
Middle Frontal Gyrus	47	R		42	36	0	3.32
Middle Frontal Gyrus	6	R	32	36	9	63	3.32
Superior Frontal Gyrus	8	R		33	24	60	3.11
Supramarginal Gyrus	40	R	34	42	-39	33	3.32
Supramarginal gyrus	40	R		36	-45	39	3.13
Insula	47	R	23	33	18	-6	3.31
Superior Temporal Gyrus	22	L	43	-66	-21	0	3.30
Superior Temporal Gyrus	22	L		-51	-18	-6	3.27
Middle Temporal Gyrus	22	L		-63	-33	3	3.04
Superior Temporal Gyrus	38	L	44	-54	9	-12	3.26
Superior Temporal Gyrus	22	L		-54	15	-3	3.23
Superior Temporal Gyrus	38	L		-48	21	-12	3.11
Superior Temporal Gyrus	42	L	11	-63	-36	18	3.25
**Positive correlation with D_4_**							
Superior Frontal Gyrus	10	R	94	9	60	33	4.28
Dorsolateral prefrontal cortex	9	R		24	54	33	4.00
Dorsolateral prefrontal cortex	9	R		9	54	45	3.86
Dorsolateral prefrontal cortex	9	L	24	-42	30	39	3.99
Cerebellum		R	55	3	-51	-27	3.82
Cerebellum		R		9	-39	-30	3.58
Middle Frontal Gyrus	6	L	12	-24	-6	66	3.67
Inferior Frontal Gyrus	45	R	11	60	27	21	3.56
Posterior parietal cortex	7	L	89	-39	-66	48	3.49
Supramarginal Gyrus	40	L		-42	-51	48	3.36
Precentral Gyrus	4	L	12	-21	-27	66	3.37
Supramarginal Gyrus	40	R	17	36	-51	33	3.34
Superior Frontal Gyrus	8	L	21	-24	36	54	3.28
Superior Frontal Gyrus	8	L		-9	48	51	3.10
Medial Frontal Gyrus	8	L		-3	51	45	3.05

*Significance threshold is p < 0.005, k = 10.*

**Table 4:** Increased activation for congruent words in comparison to congruent letters (contrast: congruent words > congruent letters).

				MNI			
Area	BA	L/R	Cluster	*x*	*y*	*z*	*t*-value
**Congruent words > congruent letters**						
Lingual Gyrus	17	R	128	21	-90	-9	5.93
Cerebellum		R	151	6	-84	-33	5.46
Cerebellum		L		-12	-78	-42	4.61
Cerebellum		L		-6	-81	-30	4.32
Inferior Occipital Gyrus	18	L	67	-33	-90	-12	4.81
Lingual Gyrus	17	L		-21	-90	-9	4.44
Inferior Occipital Gyrus	18	L		-27	-87	-21	3.56
Superior Parietal Lobule	7	L	197	-33	-69	48	4.43
Dorsolateral prefrontal cortex	46	R	66	48	42	27	4.17
Cerebellum		R	49	27	-57	-33	4.15
Cerebellum		L	58	-42	-63	-30	3.83
Cerebellum		L		-30	-51	-36	2.94
Superior Parietal Lobule	7	R	34	36	-66	45	3.71
Middle Temporal Gyrus	37	L	40	-57	-48	-12	3.69
Inferior Frontal Gyrus	47	R	23	33	24	-6	3.65
Claustrum		L	36	-30	21	0	3.61
Cerebellum		R	12	12	-75	-48	3.48
Cingulate Gyrus	32	R	11	9	24	39	3.11

*Significance threshold is p < 0.005, k = 10.*

## Discussion

The primary aim of the current study was to explore the neuro-cognitive processes associated with an IAT variant using body symptom words and neutral comparison words as targets and the dimensions “dangerous” vs. “harmless” as attributes. Since HA is characterized by automatic negative interpretations of body sensations as sign of a severe illness, the second aim of this study was to determine possible associations of both, behavioral and neural IAT effects with individual levels of HA.

In general, it appears plausible that an implicit association between symptom words and their evaluation as dangerous exists. Therefore, we expected a main effect of stronger associations between body symptoms and the attribute “dangerous” in all participants, but also hypothesized that this effect would be pronounced in subjects with higher HA. In line with the first assumption, evidence was found for an implicit association of body symptom words and the attribute dangerous. On the behavioral level, reaction times were longer for the assignment of body symptom words as harmless than for the assignment of body symptom words as dangerous. However, this was not reflected in the *D*-score that is thought to assess such implicit associations. On the neural level, stronger activation in the left DLPFC, and bilateral in the posterior parietal cortex was found in the incongruent IAT condition (compared to the incongruent letter control condition), as well as with a lower significance threshold in comparison to the congruent words [(incongruent words > incongruent letters) > (congruent words < congruent letters)], albeit the cluster size for the DLPFC activation was very small. The revealed DLPFC-activation for the incongruent condition is in line with the finding of Chee et al. (2000) and Knutson et al. (2007), who found increased activation in the DLPFC in the incongruent condition [in comparison to a letter control condition (Chee et al., 2000), respectively, a congruent word condition (Knutson et al., 2007)]. The DLPFC is a structure that is associated with working-memory processes, as well as with the modulation and inhibition of behavior (Miller and Cohen, 2001). Increased involvement of the DLPFC in the incongruent condition most likely suggests that the pairing of body symptom words with the attribute “harmless,” as well as the pairing of household words with the attribute “dangerous” introduced a need for an inhibitory process to overcome the pre-potent association. It appears noteworthy that in contrast to Chee et al. (2000), we changed the response button assignment in the incongruent control condition. Thus, in our study the stronger activation in the DLPF in the incongruent word condition in comparison to the incongruent control condition is not merely caused by a response bias, acquired in the course of the experiment, but rather represents additional necessary inhibition of the stronger association in the word condition.

In addition to the increased DLPFC activation, and also in line with the study by Chee et al. (2000), we found increased activation in the posterior parietal cortex (BA 7) bilaterally in the incongruent condition [compared to the incongruent letter control condition, as well as with a lower significance threshold in comparison to the congruent words (incongruent words > incongruent letters) > (congruent words < congruent letters)]. The posterior parietal cortex is linked to proprioception (Bolognini and Maravita, 2007), attentional processes (Ciaramelli et al., 2010), and working memory (Postle et al., 2000). The increased activation in the posterior parietal cortex can be interpreted as additional evidence for enhanced processing demands in the incongruent condition. In agreement with this assumption, we found some evidence for a positive association between activation in parietal cortex and DLPFC [(incongruent words > incongruent letters) > (congruent words < congruent letters)] and performance, as indicated by the *D*_4_ score, corroborating the importance of these areas for incongruency processing.

The second aim of the study was to investigate possible alterations in the neural processing of these negative attitudes in HA, a condition in which the protective behavior in relation to body symptoms is exaggerated. In this regard, no association was observed between HA, measured with the WI and stronger negative implicit associations on the behavioral level. However, significant associations with the WI emerged with respect to the fMRI data in the incongruent IAT condition [contrast: (incongruent words > incongruent letters), as well as with the interaction contrast: (incongruent words > incongruent letters) > (congruent words < congruent letters)]. Two of the regions that were associated with the processing of conflicting assignment of body symptom words as harmless showed increasing activation with increasing HA: The posterior parietal cortex (reaching into the superior temporal gyrus) and the DLPFC. In addition, there was increasing activation in the nucleus accumbens, reaching within the subgenual anterior cingulate, and in the cerebellum.

The correlation between HA and activation in right DLPFC and posterior parietal cortex points to the fact that the need to compensate for interference and conflict in the incongruent IAT condition and with this to inhibit pre-potent response tendencies was stronger in subjects with higher HA when forced to assign symptom words to the category harmless. The activation cluster in the posterior parietal cortex reached into the somatosensory association cortex. The somatosensory association cortex is relevant for the perception of body sensation, such as touch (Wacker et al., 2011), and is involved in pain-processing (Apkarian et al., 2005). Previous fMRI (Anders et al., 2004; Straube and Miltner, 2011) and PET (Damasio et al., 2000) studies found the somatosensory association cortex involved in the generation of emotional feelings. In addition, there is some evidence that activation in the somatosensory cortex can be modulated by emotional arousal (Rudrauf et al., 2009). Accordingly, the requirement of the incongruent task to assign body symptoms to the category harmless might have introduced more (negative) emotional feelings, and possibly increased arousal in subjects with higher HA leading to this enhanced somatosensory activation.

In addition, there was also a positive correlation between HA and brain activation in a large cluster in the ventral striatum, including the nucleus accumbens, reaching within the subgenual anterior cingulate gyrus [contrast: incongruent words > incongruent letters, as well as for a smaller cluster with the interaction contrast: (incongruent words > incongruent letters) > (congruent words < congruent letters)]. This increased activation in the nucleus accumbens points to alterations in automatic reactions to symptom words. The nucleus accumbens belongs to the limbic system and plays a role in the anticipation of reward and punishment (Stark et al., 2011). Activation in the nucleus accumbens has been theoretically (Kapur, 2003) and empirically linked to salience processing (Zink et al., 2003). Several studies found increased activity in the nucleus accumbens when stimuli were not only salient, but also behavioral- or self-relevant (Breiter et al., 2001; Zink et al., 2003; Phan et al., 2004). Therefore, possibly, the nucleus accumbens triggers a kind of warning signal indicating an incongruent behavior associated with a potentially threatening stimulus. It appears tempting to speculate that a stronger neural association between body sensations and activation in the nucleus accumbens in individuals with higher HA might represent one cause of stronger automatic negative interpretation of body sensations in this group.

Finally, neural activity in the left cerebellum during the incongruent IAT condition [compared to the incongruent letter control condition, as well as with a lower significance threshold in comparison to the congruent words (incongruent words > incongruent letters) > (congruent words < congruent letters)] was positively correlated with HA. Given previous fMRI findings on the role of the cerebellum not only regarding motor coordination but also with respect to executive functions and operations in working memory (Stoodley and Schmahmann, 2009), increased activation of the cerebellum most likely reflects the recruitment of additional processing and coordination resources for successful task performance in the incongruent IAT condition.

Taken together, the findings suggest that already in subclinical HA the automatic processing, as well as cognitive control mechanisms associated with the processing and evaluation of body symptom information are altered. Interestingly, the increased activation for incongruent words (in comparison to incongruent letters) with increasing HA was found in the right DLPFC, and right posterior parietal cortex, while the increased activation for incongruent words (in comparison to incongruent letters) over the whole group was found in the left DLPFC and in the posterior parietal cortex bilaterally. Lateralization theories for a long time suggest that the right hemisphere is predominant in the processing of (negative) affective information (Schwartz et al., 1975; Smith and Bulman-Fleming, 2005). Not disregarding that this reversal of lateralization was less pronounced for the analyses with the interaction contrast, the lateralization from the left to the right hemisphere still points to an increased demand to inhibit emotional responses to the incongruent processing of body symptom words. However, it has to be kept in mind that the association between HA and behavioral interference was not significant, either suggesting that in (subclinical) HA the reaction in the incongruent condition of the IAT is altered in terms of brain activation, but not in terms of behavior, or that the increased brain activation in HA has a compensatory effect, counteracting behavioral impairments. Other explanations are that there is simply no association between the level of HA and behavioral interference, as assessed with the *D*-score, or that our current sample size lacked power to reveal such an association. Interestingly, in line with Chee et al. (2000), but in contrast to the other studies in which neural activation associated with the IAT was investigated (Phelps et al., 2000; Knutson et al., 2006, 2007; Luo et al., 2006), we neither found activation in the amygdala during the incongruent word condition (neither in comparison to the incongruent letter control condition, nor in the interaction contrast), nor a correlation between HA and amygdala activation. To our knowledge, the studies in which amygdala activation was found, were all related to the implicit assessment of either racial, or gender stereotypes. However, it was shown in a lesion study that the amygdala is not necessary for the existence of an implicit racial bias, as measured by the IAT (Phelps et al., 2003). Beer et al. (2008) concluded that the amygdala seems to reflect in-group favoritism. Thus, concluding from the current state of knowledge from existing functional imaging studies applying an IAT, the amygdala seems not to be an essential region for the representation of implicit associations, but to be specifically involved in implicit associations concerning stereotypes. However, it remains open whether the amygdala would be involved when implicit associations of symptom words were investigated in patients with pathological HA, or could be revealed with a larger sample.

Several limitations to our study are noteworthy. First of all, our findings are based on a sample of individuals with rather low to moderate levels of HA. Further clinical studies with patients suffering from pathological HA are necessary to confirm and validate the current findings. In addition to implicit associations of attitudes toward body symptoms, future studies should include measures of explicit ratings of body symptoms to test possible associations between implicit and explicit evaluations and attitudes. In addition, an inspection of the RT and accuracy data depending on the stimulus type (i.e., words vs. letters) revealed that different speed-accuracy tradeoffs existed: While both accuracy and reaction times were lower in the incongruent condition, this effect was pronounced for the reaction times in the word condition, and for the accuracy data in the letter condition. It is noteworthy that in such a case, the IAT effect is blurred in the *D*_4_ measure, because errors are transferred into RT units (i.e., by adding an error penalty of 600 ms), and explains that the IAT-effect was only found in the RT-data and not in the *D*_4_ score. However, a recent study of our group revealed that patients with pathological HA show response slowing in the incongruent condition, but do not have altered implicit associations as assessed by the *D*-score (Witthöft et al., 2015). This finding suggests a dissociation between implicit associations, as assessed with the *D*-score, and general response slowing also in pathological HA. In addition, increased activation in the incongruent in comparison to the congruent condition was only revealed when the significance threshold was lowered. The same pattern occurred for the association with IAT performance. Replication of this study with a larger sample size, or a group of patients suffering from pathological HA will reveal whether the difference between the congruent and the incongruent condition is only small, or whether the current study lacked sufficient power to reliably reveal the difference.

## Conclusion

In general, the current findings regarding the neural processes associated with an symptom word IAT replicate previous findings that have been reported with the classic insect-flower IAT (Chee et al., 2000), as well as with IATs assessing gender and racial attitudes (Beer et al., 2008). In line with prior findings, the DLPFC and posterior parietal cortex showed increased activity in the incongruent IAT condition (chiefly in comparison to the control condition, and less pronounced also in comparison to the congruent IAT condition). This activation and the association with activation in the NAcc were moderated by individual levels of HA. These results point to alterations in implicit as well as in control mechanisms in the processing of body symptom words in HA. Since significant correlations with neural activation and HA emerged only in the incongruent IAT condition, i.e., during conflicts between preexisting and habitual associations and required responses, subclinical HA appears to be associated particularly with problems in overriding symptom-dangerous associations in memory which might predispose these individuals to develop pathological HA later in life.

## Author Contributions

Each authors read and approved the final version of the manuscript. DM: data acquisition, data analyses, and draft. MW: experimental design, data analyses, and draft. JB: study design and experimental design. TK: data acquisition and data analyses. JO: programming, data acquisition, and data analyses. FR: study design and experimental design. CD: experimental design and data analyses.

## Conflict of Interest Statement

The authors declare that the research was conducted in the absence of any commercial or financial relationships that could be construed as a potential conflict of interest.
